# Fourth Report of the Resident-Physician of the County of Middlesex Pauper Lunatic Asylum at Hanwell, October 1st, 1842

**Published:** 1843-01

**Authors:** 


### Art. XIX.

*Fourth Report of the Resident-Physician of the county of Middlesex Pauper Lunatic Asylum at Hanwell, October* 1*st*, 1842. 8vo, pp. 49.

This is another of those admirable documents to which we have already more than once called the attention of our readers, and which must ever give to the name of Dr. Conolly and of Hanwell a preeminent position in all future accounts of the treatment of insane persons. To him unquestionably belongs the glory, not indeed of inventing or first applying the practice of non-restraint in the treatment of insanity, but of reducing it to a system, enforcing it on a large scale against all sorts of prejudices, and persevering in its exclusive use, until the eyes of the whole world were opened to its perfect practicability and its infinite superiority over the ancient system of harshness and confinement. The demonstration of this fact by Dr. Conolly at Hanwell, has had the important result of causing the same rational system, in a greater or less degree, to be adopted at almost all other British asylums, so that the treatment of lunatics by bodily restraint may now be said to be the exception, not the rule, in this country. The following extracts from the report before us record this most gratifying fact:

"In the mean time, the *entire disuse* of restraints is making a constant progress, every step of which is rendered secure by careful observation. In the Lincoln Asylum, after many difficulties, the plan, which originated there, seems at length to be completely established. At Northampton it has been steadily and zealously persevered in. It has been ably carried into effect at Stafford. Since the recent appointment of Dr. Anderson to the asylum attached to the Royal Navy Hospital at Haslar, it has been adopted with the most entire success, notwithstanding some peculiar impediments arising from the plan of the building; and it has been conjoined with several other beneficial alterations, under the high sanction of Sir William Burnett, the head of the medical department of the navy. The latest report of the Gloucester Asylum, an institution always distinguished by the early adoption as well as by the suggestion of various improvement, states that the *total disuse* of all mechanical restraints has been sanctioned by the visiting committee, and that 'all the patients are as securely managed, and are governed with much less difficulty and disturbance, without than with mechanical assistance.' In the Lancaster Asylum, containing nearly 600 patients, no restraints have been employed for seventeen months: walls have been lowered, iron bars removed, the means of exercise and recreation increased, and all the parts of an improved system of management carried into operation, notwithstanding some peculiar difficulties.

"In the latest report of the Edinburgh Lunatic Asylum, the physician says, 'For eighteen months I have not considered it necessary to use personal restraint on any occasion during the day. In one case (a suicidal patient), I have been reluctantly compelled to make use of it during the night; but with a larger staff of attendants, or a building constructed on the plan of the new establishment, I would have been enabled to dispense with it even in that instance.' The latest report of the Dundee Asylum contains the following brief but satisfactory statement: 'There is not one patient restrained personally by mechanical means in this asylum.' In several asylums, where as yet it has not been found practicable to discontinue restraints entirely, they are spoken of as being seldom resorted to. At York, Ipswich, Dumfries, Belfast, Clonmel, and in the asylums of Worcester, Bloomingdale, and Massachusetts, in the United States, this testimony has been distinctly given. The circumstance of the foundation-stone of a new asylum now erecting near Glasgow, being made the record of the intention of the directors, that no bodily mechanical restraint shall ever be used in it, is remarkable in the history of asylums; and is no doubt to be ascribed to the opinions formed by Dr. Hutcheson, after a patient and candid inquiry into the merits of this system of treatment." (pp. 43-4.)

The effect of the new system on the patients at Hanwell is thus described by Dr. Conolly, after an experience of more than three years:

"The annual reports presented by the resident-physician in 1839, 1840, and 1841, contain the details of a plan adopted by him from the Lincoln Asylum, and persevered in, with such modifications as experience suggested, with the sanction of the visiting justices, to dispense, in the treatment of the insane, with all the ancient bodily restraints. The difficulties attending the commencement of the undertaking, its progress, and its eventual success, have been already related in those reports without disguise, and, it is believed, without exaggeration. The resident-physician has now but the agreeable task of recording, that time and patience, and the zealous cooperation of all the officers of the asylum, have enabled him to overcome many obstacles, and have confirmed him in a belief, at first encouraged with much diffidence, but now established beyond the likelihood of ever being overthrown, that the management of a large asylum is not only practicable without the application of bodily coercion to the patients, but that, after the total disuse of such a method of control, the whole character of an asylum undergoes a gradual and beneficial change.

"A long indisposition, attended with a mortifying interruption of your physician's active duties, has, by converting him into little more than a spectator of what was taking place, at least enabled him to exercise the calmer observation of a bystander; and his return to the asylum, after the absence accorded to him by the kindness of the visiting justices for the recovery of his health, gave him an opportunity, by the strong impressions incidental to a return to so extraordinary a scene, to appreciate, perhaps more justly than he could otherwise have done, the general results of a system now three years in undisturbed operation, excluding, as much as possible, every cause of physical and mental uneasiness. The appearance and general state of the patients in the wards, or when taking exercise; when engaged with in-door amusements, or when assembled at dinner or supper; when at work in the various departments of industry connected with the institution, or when attending divine service; the order, activity, and cheerfulness pervading the asylum by day, and the tranquillity of the whole house by night, are all indications that the general management of so many disordered minds is productive of those salutary effects which are the object of all management.

"The impression produced on patients newly admitted to the asylum is also strongly indicative of the general character of the place being favorable to curative endeavours. Their wildness and irregularities often rapidly subside, and their habits conform to the general order and the decorous routine so remarkable in the majority of their fellow-patients. The continued operation of a tranquillizing system has produced effects even on the character and manners, and, as it would seem, on the disposition of not a few of the old and incurable patients; several of whom, formerly accustomed to meet the officers with endless complaints, seem now to have lost their fretfulness, and to be satisfied and content. Accidents, anxieties, and agitations must always be incidental to any house in which all forms and varieties of mental disturbance and disease are accumulated; but the resident physician believes that all the officers of asylums who are experienced in both methods of treatment, have found, or will find, that the liberation of their patients from restraints has lessened the frequency of accidents, and diminished the anxieties and agitations of those having the charge of them; so that even the various contrivances at first required for the prevention of evils and inconveniences formerly opposed by restraints, as strong dresses, seclusions, and window guards, become less required.

"It continues occasionally to happen that patients are brought to the asylum in severe restraints. These are invariably removed at once; and they are never put on again: yet from this immediate and systematic liberation not one important accident, scarcely any inconvenience, has arisen. Every admission more strongly manifests the importance of regulating all the circumstances connected with the arrival and reception of a patient, with a strict regard to reconciling the new comer to the asylum. The only instance during the last year in which the attendants were set at open defiance, and one of them was severely hurt by a patient just received, occurred in the instance of a male patient, who was so quiet before his arrival, that he had been entrusted to drive the carriage which conveyed him and others during a part of the journey; but he had been told that the other patients only were to remain, and on discovering this foolish deception, he became, for a day or two, unruly. He afterwards behaved extremely well.

"The state of mind of a patient of ordinary sensibility, on arriving at the gates of a lunatic asylum, is usually somewhat agitated. It is then, amidst the fears and distress of the sufferer, to whom all is new and strange, that confidence is to be gained, and the first steps of successful moral treatment are to be taken. The manner in which new patients are addressed, the attendants to whom they are confided, the personal interest taken in them by the officers, the wards in which they are placed, the employment assigned to them, are all matters of great consequence; not only as allaying immediate anguish in many cases, but as exercising an influence over every curable patient from the hour of reception to the hour in which they leave the asylum." (pp. 33-6.)

After detailing the very interesting particulars of some cases strikingly illustrative of the speedy influence of the new system, Dr. Conolly continues:

"The difficulty of controlling a violent lunatic in a private house, or in a workhouse, might be admitted as some excuse for the marks of cords and fetters visible on some of the patients; but others were so harmless, and even so helpless, that their previous coercion could only be ascribed to the unfortunate habit of treating every lunatic with severity. So long as the asylum, large as it is, is yet inadequate to the reception of all the pauper lunatics of this populous county, there will still be cause to regret that many patients are transmitted to the asylum whom either the long continuance of their insanity, or exposure to previous injudicious management, or both united, have brought into a state unfavorable to the cure or even to any remarkable mitigation of their malady. At present it sometimes happens that five or six patients are admitted in one day, all brought from some other asylum, and in not one of whose cases there is the slightest hope of amendment; and there is no doubt that there are, at all times, many recent cases in the county which are gradually falling into the irremediable condition in which they will at length be sent to Hanwell.

"There is, however, great reason to hope that the injudicious practices above alluded to, by which the early and most important period of the malady is too often wasted, will soon be entirely unknown in all asylums, public and private. The reports of many public asylums contain statements and observations strongly indicative of a general and progressive improvement, directed by an increasing conviction, confirmed by all intimate experience with insane persons, that in a large majority of cases of insanity the powers of observation are active, and the understanding has a considerable range of exercise; whilst the affections exist as warmly, and the sensibility is as acute as in a state of perfect mental health. Instead, therefore, of the majority of insane persons being now consigned to the chance of cruelty or oblivion, the utmost care is taken to act on what remains of intellect and feeling in each case; so as to direct the impaired faculties of the understanding, if not always usefully, at least safely, and at the same time to cherish and govern the affections by all the resources of compassionate protection. Such, unquestionably, are the general principles by which the treatment of the insane is regulated at the present period, variously carried into effect in various asylums. The results are daily developing themselves to all daily observers, and can scarcely yet be fully known and appreciated; but every year is affording new and satisfactory proofs that the principles are not dangerous or delusive, but founded in reason and fertile in advantages. Neglect and disregard, violence and intimidation, aided by all the devices of mechanical restraints, are everywhere disappearing; and everywhere, as they disappear, the application of all the powers and influences of sound mind to the recovery of mind impaired takes the place of them. The consequences may not be that a much greater number of perfect recoveries are effected, for recovery is impossible in a majority of cases of insanity; but the actual number of the insane thus kept in the living and intellectual world, and enjoying a great share of happiness, is immensely increased.

"Insanity, thus treated, undergoes great if not unexpected modifications, and the wards of lunatic asylums no longer illustrate the harrowing description of their former state. Mania, not exasperated by severity, and melancholia, not deepened by the want of all ordinary consolations, lose the exaggerated character in which they were formerly beheld. Hope takes the place of fear; serenity is substituted for discontent, and the mind is left in a condition favorable to every impression likely to call forth salutary efforts. A chance is thus afforded, to every impaired mind, of recovery to an extent only limited by causes which no human art can remove." (pp. 40-2.)

Under the heads of Entertainments and Instruction to the Patients, the following most interesting facts are stated:

"The entertainments given to the patients during the year have been more numerously attended, and more varied in character, than in former years. On New Year's eve, about 300 female patients partook of their usual entertainment in one of the wards, which was fitted up with evergreens. Among the patients at this party there were nineteen who were formerly always, or almost always, in restraints. During the summer, the matron's birthday was celebrated by a still larger party of the female patients, who drank tea, danced, and played various active games in the field in front of the left wing.

"Until this year, the male patients had not been indulged with any similar entertainments; but in January last, rather more than 200 of them, including all who are employed in any way, had coffee and cake in the evening, then amused themselves by singing, dancing, and music, some of the attendants giving able assistance, until eight o'clock, when they had an excellent hot supper (roast beef and apple pies), with one pint of beer, some tobacco, and a new pipe for each patient desirous of smoking. The scene presented by them was one of the utmost cheerfulness and good humour. All behaved well; thus showing that the male patients are no less capable than the female patients had been found to be, of partaking of moderate gaiety without any neglect of decorum. Several banners were hung over the tables, made by one of the patients, and containing mottoes complimentary to the magistrates and to the officers. One of them was inscribed, 'To our noble selves.' The wards were all visited late at night, and only one or two patients were noisy, and these had not been at the party. A few days afterward a grave elderly Scotch patient, who lays claim to titles and wealth, expressed a wish to the house-surgeon that he would give 'those rebels' who slept in the same room with him, 'a supper every night;' for that whereas they were usually singing and noisy, they were on that particular night perfectly quiet.

"During the summer, about the same number of the male patients had an entertainment out of doors, for which occasion a band of music was organized, consisting partly of patients. One of the most violent and troublesome men in the house, an old soldier and bugler, beat the brass drum very correctly, although rather more vehemently than was required."

"To look upon these entertainments as mere occasions of display, would be to degrade them from their principal use. Their effect is to cheer and console the depressed, as manifestations of the consideration felt for them, and the desire entertained for their happiness, and to interrupt the unhappy thoughts of the more disturbed with the associations of innocent diversion and joyousness. Such evenings are known to be looked forward to with pleasing anticipations for several weeks, and the patients join in the bustle of preparation for them with alacrity and cheerfulness. The happy assembling, the delight evinced by many during the hours of the entertainment, the gratified expressions of the patients on the breaking up of the party, their orderly and good-humoured departure from the scene of this simple gaiety, leave the unaccustomed spectator impressed with wonder, and those most familiar with such a scene filled with the emotions naturally arising from the view of so much happiness, created by the mere exercise of kindness, in mansions thought to be dedicate only to scenes of suffering and woe.

"If even these effects were merely transient, their advantage might be deemed inconsiderable. But they are not so. For weeks afterward the patients retain an agreeable recollection of their 'pleasant party.' The little indulgences then permitted are found to blend themselves with all the best parts of moral management, and to contribute to secure the confidence and the affection of the insane. The gratitude thus created becomes a bond of great power; for the patients, in general, fully appreciate all that is done, not only to protect them from suffering, but to impart positive comfort and enjoyment to them." (pp. 26-9.)

"By the kind exertions of the chaplain, an attempt has recently been made, with the resident-physician's cordial concurrence, to ascertain the practicability of imparting some general as well as religious instruction to a certain number of the male and female patients, of various degrees of capacity. This has been done by the establishment of reading classes, to which such patients were admitted as appeared to be capable of reading and understanding the simple books placed in their hands, or as were particularly desirous of joining a class.

"They attended about twice a week, for an hour, in classes varying from six to fourteen in number. Each patient read a few passages in turn, and the subject of the reading, which was always of a kind easy to be understood by those who were in the class, was rendered clearer or more amusing by the help of pictures, or a map, or a familiar explanation. It was interesting to observe the effects of these efforts on the patients. An elderly male patient was at first remarked always to attend without his spectacles, which he alleged as an excuse for not taking his part in the reading; but after a few attendances, he seemed to become less diffident or less distrustful of himself, and when he had finished a passage he expressed great satisfaction at his own success. A female patient evinced, at first, a similar reluctance; but afterward read willingly to the chaplain when in her own ward. Another patient, an epileptic woman, at first articulated with difficulty; but appeared to improve at each successive reading. The patients frequently corrected the reading of those who read incorrectly, and their interference was submitted to with perfect good humour. One patient, who could not be persuaded to read, was interested in the explanation of what was read; and all seemed to be pleased with the map of Palestine and with the illustrative pictures.

"About 120 patients on each side of the asylum were thought by the chaplain, after careful inquiry, to be capable of attending such classes with more or less advantage: but, among those who shared his attention in the course of this interesting and benevolent experiment, there were some of very feeble understanding and unacquainted even with the alphabet. Yet some of these evinced pleasure when an attempt was made to teach them. One poor epileptic boy learned all the letters in about ten lessons. Others, of very unpromising appearance, were found able to repeat more or less of the Lord's prayer; and they did this with folded hands, and in a manner showing that the recollection of some early lessons was not wholly effaced.

"All the experience obtained in these attempts was, in short, of a nature to encourage perseverance in, and an extension of them; and to make it appear probable that most of the aids of juvenile, or at least of infant education, might be introduced into lunatic asylums for the poorest patients, with results not only singular in their character but generally beneficial to the patients, and in accordance with all that is endeavoured to effect for them by other means." (p. 30-1.)

It is impossible to read these beautiful passages without deep emotion, fraught as they are with the lessons of a pure and genuine philanthropy, and recording one of the most glorious, because one of the serenest and most peaceful triumphs of humanity. Well does this Report vindicate the claim of Hanwell to the motto which we have assigned to her in another page:

"Hier wird durch die That bewiesen, was der Mensch ueber den Menschen durch das Menschliche vermag."

---

